# Polymorphism-Aware Species Trees with Advanced Mutation Models, Bootstrap, and Rate Heterogeneity

**DOI:** 10.1093/molbev/msz043

**Published:** 2019-03-02

**Authors:** Dominik Schrempf, Bui Quang Minh, Arndt von Haeseler, Carolin Kosiol

**Affiliations:** 1 Department of Biological Physics, Eötvös Loránd University, Budapest, Hungary; 2 Centre for Biological Diversity, University of St Andrews, St Andrews, United Kingdom; 3 Center for Integrative Bioinformatics Vienna, Max F. Perutz Laboratories, University of Vienna, Medical University of Vienna, Vienna, Austria; 4 Ecology and Evolution, Research School of Biology, Australian National University, Canberra, ACT, Australia; 5 Research School of Computer Science, Australian National University, Canberra, ACT, Australia; 6 Bioinformatics and Computational Biology, Faculty of Computer Science, University of Vienna, Vienna, Austria; 7 Institut für Populationsgenetik, Vetmeduni Vienna, Austria

**Keywords:** incomplete lineage sorting, species tree, phylogenetics, polymorphism-aware phylogenetic model, boundary mutation model

## Abstract

Molecular phylogenetics has neglected polymorphisms within present and ancestral populations for a long time. Recently, multispecies coalescent based methods have increased in popularity, however, their application is limited to a small number of species and individuals. We introduced a polymorphism-aware phylogenetic model (PoMo), which overcomes this limitation and scales well with the increasing amount of sequence data whereas accounting for present and ancestral polymorphisms. PoMo circumvents handling of gene trees and directly infers species trees from allele frequency data. Here, we extend the PoMo implementation in IQ-TREE and integrate search for the statistically best-fit mutation model, the ability to infer mutation rate variation across sites, and assessment of branch support values. We exemplify an analysis of a hundred species with ten haploid individuals each, showing that PoMo can perform inference on large data sets. While PoMo is more accurate than standard substitution models applied to concatenated alignments, it is almost as fast. We also provide bmm-simulate, a software package that allows simulation of sequences evolving under PoMo. The new options consolidate the value of PoMo for phylogenetic analyses with population data.

## Introduction

Molecular phylogenetics seeks to estimate evolutionary relationships of species depicted as species trees by modeling the change and development of hereditary sequences. Established methods (e.g., Tavaré 1986; Yang 2006) that neglect molecular variation on the population level suffer from incongruencies between genomic regions which may arise due to incomplete lineage sorting (ILS, Maddison 1997; Knowles 2009). The effect of ILS becomes large if the considered species are closely related or if internal branches of the species tree are short when measured in number of generations divided by the effective population size (Pamilo and Nei 1988). Short branches, especially when appearing in caterpillar-like topologies may lead to statistical inconsistency (Degnan and Rosenberg 2009; Degnan 2013) of approaches such as concatenation (Gadagkar et al. 2005), where sequences for different genes of the same individual are joined to form one overall alignment.

Consequently, development of phylogenetic methods has increasingly focused on explicit modeling of population genetic effects (Leaché and Oaks 2017). Most methods employ the multispecies coalescent model (Rannala and Yang 2003) to reconcile gene trees, that is, the evolutionary histories of genes, with the species tree. The species tree is either jointly estimated with the gene trees (e.g., Drummond and Rambaut 2007; Liu 2008; Heled and Drummond 2010) or reconstructed from previously estimated gene trees (e.g., Liu et al. 2010; Mirarab et al. 2014).

We have recently proposed a polymorphism-aware phylogenetic model (PoMo, De Maio et al. 2015; Schrempf et al. 2016) for estimating species trees from genome-wide data for up to dozens of species with multiple individuals each, which bypasses the computational burden of estimating gene trees. PoMo can be viewed as an extension of classical substitution models which additionally considers polymorphisms. Present as well as ancestral polymorphisms are described along a species tree by separating mutation and genetic drift in a population genetic framework that we have termed multivariate boundary mutation model (Vogl and Clemente 2012; Schrempf and Hobolth 2017). The substitution model (e.g., HKY model, Hasegawa et al. 1985) extended by the multivariate boundary mutation model is referred to as mutation model, because frequency changes are considered separately. PoMo improves estimation of mutation parameters and species trees compared with established methods such as concatenation when ILS has blurred the phylogenetic signal. Extensive evaluations showed good performance of PoMo against other methods (De Maio et al. 2015; Schrempf et al. 2016). Here, we extend our implementation into the maximum likelihood software IQ-TREE (Nguyen et al. 2015), referred to as IQ-TREE-PoMo, which includes several new features as detailed in the next section.

## New Approaches

### Big Data

In contrast to the multispecies coalescent model, PoMo scales much better with the number of analyzed species. The performance of IQ-TREE PoMo was tested by applying it to simulated multiple sequence alignments (MSA) of a length of up to 1 million nucleotides, and 100 species with ten sampled individuals each corresponding to gene trees with 1,000 leaves (see Materials and Methods).

### Simulator

We developed and released a software package to generate sequences evolved under various boundary mutation models (bmms), in particular PoMos, called bmm-simulate (https://github.com/pomo-dev/bmm-simulate; last accessed March 11, 2019). Given a species tree, the simulator directly generates polymorphic sequences using the discrete multivariate boundary mutation model with mutation rate heterogeneity (see below). bmm-simulate enables exact assessment of IQ-TREE-PoMo because the same model is used for simulation and inference, and complements existing simulators based on the multispecies coalescent model, which we have used in this and in previous studies. Furthermore, inference methods based on the multispecies coalescent model could be validated against data simulated with bmm-simulate.

### Advanced Models

Heterogeneity of rates across sites is a consequence of varying mutation and fixation rates. If rate heterogeneity is not taken into account, sequence distance is underestimated and phylogenetic analyses may suffer from long branch attraction artifacts (Yang 2006, p.19).

Therefore, we developed new theory for PoMo to incorporate Γ distributed mutation rate heterogeneity across sites (Yang 1994). The new theory is necessary as only the mutation rates are heterogeneous across sites, whereas drift rates stay constant for our model (see Materials and Methods). A simulation study with bmm-simulate showed high accuracy when estimating the Γ shape parameter and other parameters on trees with twelve species.

Furthermore, species tree search can be performed with fixed parameters. Manual specification of parameters is useful when detailed knowledge is available or when likelihoods are compared between different analyses. In practice, we often do not have total control over the number of individuals in the population that are sampled and sequenced (e.g., sequences are retrieved from existing databases, resequencing runs failed or are simply too costly even for as few as ten individuals). We therefore present two strategies, called weighted binomial and weighted hypergeometric sampling, to initialize the likelihoods of the PoMo states at the leaves of the trees, and to account for this missing data problem.

### ModelFinder

Model selection, which includes finding the best-fit mutation model as well as a model of mutation rate heterogeneity across sites, is crucial when performing molecular phylogenetic analyses. The improved implementation of PoMo allows for highly flexible polymorphism-aware phylogenetic analyses in that the most suitable evolutionary model for the data at hand can be automatically determined using statistical model search with ModelFinder (Kalyaanamoorthy et al. 2017). ModelFinder is a model selection framework in IQ-TREE which has now been made available for IQ-TREE-PoMo. The Bayesian (Schwarz 1978) or the Akaike (Akaike 1973) information criterion are employed to determine the best-fit model.

### Bootstrapping

IQ-TREE-PoMo is fast enough to allow for bootstrapping (Efron 1979; Felsenstein 1985) on large data sets. Evaluation of branch support values using the branch-wise approximate likelihood ratio test (SH-aLRT, Guindon et al. 2010) as well as standard nonparametric bootstrap (Efron 1979) and ultrafast bootstrap (UFBoot2, Hoang et al. 2018) is now possible. The ability of bootstrapping was tested on a data set of great ape genomes (see Materials and Methods, Prado-Martinez et al. 2013).

## Results

### Big Data

**Fig. 1.**
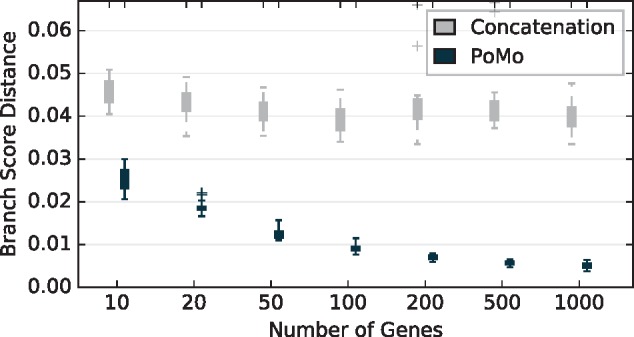
Branch score distance of concatenation approach and IQ-TREE-PoMo with *N *=* *10 and weighted binomial sampling for Yule trees with 100 species and ten individuals each. The tree height measured in coalescent units is $6N_{e}$, where *N_e_* is the effective population size. The HKY model was used for both inference methods. The heterozygosity is $\theta_{W}=0.005$ per site. Each gene spans 1,000 sites. The error bars are standard deviations of ten replicate analyses.

The phylogenetic analysis of the simulated MSAs of 100 species with ten sampled individuals each shows that consideration of heterozygosity improves estimates considerably compared with the standard concatenation approach. Branch score distance (fig. 1; Kuhner and Felsenstein 1994), as well as Robinson–Foulds distance (supplementary fig. S1, Supplementary Material online; Robinson and Foulds 1981) strongly decrease, especially when sufficient data are available. Remarkably, IQ-TREE-PoMo already outperforms concatenation for as little as ten genes (10,000 sites) and exhibits branch score distances around ten times more accurate when 1,000 genes (one million sites) are available. Further, the progression of branch score distance for the concatenation method with increasing amount of data is not monotonically decreasing. The average run time (wall-clock time) of IQ-TREE-PoMo for 1,000 genes which corresponds to a sequence length of one million sites is 12.3 ± 0.7 h on a 2.6 GHz processor with 16 physical cores (Intel Xeon CPU E5-2650 v2 @ 2.60 GHz). The concatenation method is approximately six times faster (2±0.25 h).

### Advanced Models

**Fig. 2.**
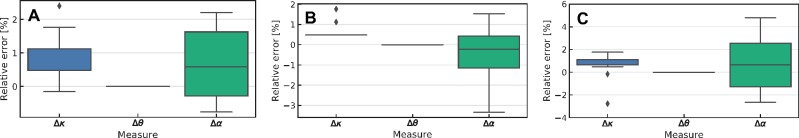
Relative errors of the transition to transversion ratio Δκ, the heterozygosity Δθ, and the shape parameter of the Γ distributed mutation rate heterogeneity Δα. The true shape parameter is α=0.3 (*A*), α=1.0 (*B*), and α=5.0 (*C*), respectively.

We assess the accuracy of estimating rate heterogeneity by focusing on the shape parameter *α* of the Γ distribution. The relative error (difference between true and estimated value in percent) of the shape parameter *α* for ten replicate analyses is usually within two percent (see fig. 2 for α=0.3, 1.0, and 5.0, respectively) but can be higher for more extreme *α* values (≈20 and ≈400 percent for α=0.1, and 10, respectively; cf. supplementary figs. S2–S4, Supplementary Material online which also show the relative error of the variance of the associated Γ distributions). The relative errors in terms of branch score distance and the transition to transversion ratio parameter of the HKY mutation model *κ* are mostly below 0.5 and one percent, respectively.

**Fig. 3.**
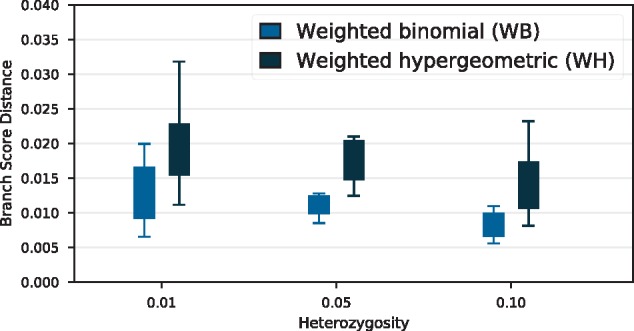
Branch score distance of weighted binomial and weighted hypergeometric sampling for Yule trees of height $3N_{e}$ with twelve species and ten individuals each. Heterozygosity varies between 0.01 and 0.1.

The heterozygosity *θ* determines the level of polymorphism present in the species. We tested the accuracy of PoMo with increasing heterozygosity because for high values of *θ* the model assumption of having boundary mutations only is violated. We observe low errors in branch score distance for heterozygosity values up to θ=0.1 in analyses of species trees with twelve species with ten individuals each and a tree height of $3N_{e}$ (fig. 3).

For the case of uneven sampling of individuals of the populations, we tested two different strategies to account for the missing data in the poorly sampled populations. We found that weighted binomial sampling outperforms weighted hypergeometric sampling (see Materials and Methods).

### ModelFinder and Bootstrapping

**Fig. 4.**
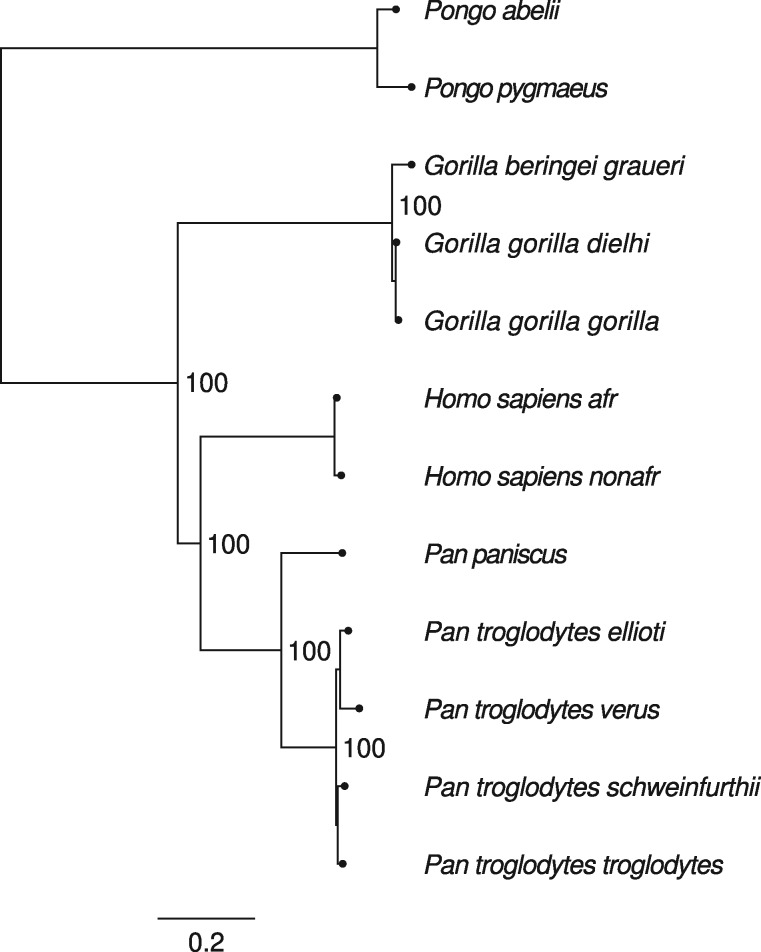
Phylogeny inferred from primate data (Prado-Martinez et al. 2013). Both, UFBoot2 (Hoang et al. 2018) and SH-aLRT (Guindon et al.2010) branch support tests evaluated to hundred percent support values.

Model selection and bootstrapping were tested on data of 12 great ape species (see Materials and Methods). For PoMo, the GTR (Tavaré 1986) mutation model with Γ rate heterogeneity was determined to be the best fitting model. The inferred Γ shape parameter is 1.26. The estimated phylogeny (fig. 4) confirms previous results (Schrempf et al. 2016). UFBoot2 and SH-aLRT both report 100 percent support for all branches.

## Discussion

The analysis of data from 100 species underlines that IQ-TREE-PoMo is fast and scales well with increasing number of species. The chosen species tree exhibits a significant amount of ILS, and hence, it is expected that our method which accounts for polymorphisms has higher accuracy than concatenation. In contrast to the concatenation method, which exhibits statistical inconsistency (fig. 1) as previously reported by Degnan and Rosenberg (2009) and Degnan (2013), the branch score distance of IQ-TREE-PoMo continuously decreases when more data are analyzed (fig. 1).

Further, it is now possible to infer parameters of more advanced sequence evolution models with IQ-TREE-PoMo. When modeling mutation rate heterogeneity, the shape parameter *α* of the Γ distribution is recovered with relative errors below two percent for 0.3≤α≤5.0. The inferred value of α=1.26 for the great ape data set lies well in this range. We observe a slight overestimation of the shape parameter, when a significant amount of sites evolves extremely slowly and is nearly invariant (α≤0.1, supplementary fig. S2, Supplementary Material online). Similarly and as expected, when the mutation rate distribution is homogeneous (α≥10.0), the variability of estimated shape parameters is higher (supplementary fig. S4, Supplementary Material online).

We decided against implementing the invariant sites model. The reason is that polymorphisms are the consequence of strictly positive mutation rates. When mutation rates are zero, polymorphic states have a stationary frequency of zero. This means that observed polymorphic sites cannot be meaningfully assigned to any model state. Note that this is conceptually different from observing a variable site pattern when using a classical substitution model with invariant sites, because then each character has a nonzero stationary frequency, only the variable site pattern has a likelihood of zero. The identity matrix could also be used as transition probability matrix for invariant sites with PoMo, but this would mean that two boundary states with different alleles have the same “distance” as two neighboring states in the PoMo state space. Besides, we strongly believe that, if the data exhibit nearly invariant sites, those are sufficiently covered by Γ distributed mutation rate heterogeneity with low shape parameters.

As Mallo (2017) reported increased errors of the estimates of PoMo for high heterozygosity values up to θ=0.05, we tested the robustness of our approach in this respect. We find that the accuracy of IQ-TREE-PoMo with respect to branch score distance increases with heterozygosity values up to θ=0.1, which is well above the observed value in primates (θ∼0.001, Prado-Martinez et al. 2013) and most other organisms (except, Lynch et al. 2016).

With respect to sampling strategies (see Materials and Methods), weighted binomial sampling has been repeatedly observed to be the most accurate (e.g., fig. 3) and is used by default. However, weighted binomial sampling has a minor disadvantage as heterozygosity is overestimated compared with the data. When observing polymorphic sites, the likelihood of monomorphic states is initialized to zero. But when observing monomorphic sites, the likelihood of polymorphic states is initialized to nonzero values leading to an increased level of heterozygosity. Weighted hypergeometric sampling reduces the overestimation of heterozygosity (and exactly retains the level of heterozygosity when the number of samples is equal to the number of PoMo frequency bins) but has other disadvantages such as undefined behavior when the number of samples at a single site and leaf is larger than the number of PoMo frequency bins.

Further, as more advanced mutation models are now available, the choice of the statistically most adequate mutation model is of major importance. ModelFinder does not require user input and is fast. The likelihood improvement when accounting for mutation rate heterogeneity is also tested for and, consequently, the most appropriate mutation rate heterogeneity model is reported and automatically used.

For the great ape data set, the best-fit model for PoMo coincides with results from concatenation methods and from the phylogenetic analysis in the original paper (Prado-Martinez et al. 2013). In the latter case, not only the model choice coincides, but also the topology, which is a further confirmation of the validity of PoMo.

Additionally, assessment of branch support values is now possible with normal bootstrap, UFBoot2 and likelihood ratio based tests (SH-aLRT). Branch support values for the primate data are high because 2.8 million sites are considered. This is not surprising and emphasizes the high confidence of the results, but not necessarily low systematic error—a general problem of bootstrap analyses on large data sets. However, especially for smaller data sets, assessment of branch support values will be highly useful. In the future, we will investigate the utility of the jackknifing options already available in IQ-TREE in the context of PoMo species tree estimation from very large data sets.

Overall, we provide important extensions to further establish the use of polymorphism-aware models in phylogenetics. Although we observe that PoMo is robust with respect to higher levels of heterozygosity, an additional sensitivity analysis under balancing selection may be useful. In the future, we would also like to implement probability distribution free rate category models (Kalyaanamoorthy et al. 2017). Furthermore, by enabling nonreversible mutation models consistent with heterogeneous mutation rates (Lie–Markov models, Woodhams et al. 2015), we envisage the possibility of quantifying the deviations of the evolutionary process from reversibility when assuming stationarity.

IQ-TREE offers an extensive set of mutation models, allows usage of new model selection methods such as ModelFinder, and comes with other benefits such as the ability to resume analyses (checkpointing), or carefully designed parallelization techniques. In conclusion, PoMo has evolved to be a mature, well-tested and flexible method to perform phylogenetic inference with population data.

## Materials and Methods

### PoMo

A detailed description of PoMo and the discrete multivariate boundary mutation model can be found in Schrempf et al. (2016) and Schrempf and Hobolth (2017), respectively. PoMo is a time-continuous Markov process modeling sequence evolution along a species tree. Sites are assumed to be independent (composite likelihood, free recombination). At each site, not only evolution of a single character a∈A (e.g., A={A,C,G,T}, |A|=4) of the reference genome is considered but rather the evolution of the collective characters of a population of genomes.

Actual populations are big in size and direct treatment is not feasible. For neutral evolution, effective population size is confined with mutation rates. It is possible to scale down the effective population size to a small value while scaling up mutation rates such that the overall dynamics remain unchanged. We take advantage of this property by choosing a rather small number of collected characters *N* of the multivariate boundary mutation model. Consequently, the parameter *N* should be interpreted as a discretization parameter (not to be confused with effective population size) describing the number of bins that allele frequencies can fall into.

The rates of frequency shifts are determined using the time-continuous Moran process (Moran 1958). PoMo assumes that drift removes variation fast, and mutations are disallowed when more than one allele is present in the population. Hence, the population can either be monomorphic for an allele a∈A or polymorphic for two alleles a,b∈A with counts *i* and (N−i), respectively. The monomorphic and polymorphic states of the multivariate boundary mutation model are denoted by {*a*} and {ia|(N−i)b}, a≠b, respectively. Disallowing mutations when the population is polymorphic is a good approximation as long as the heterozygosity is below a value of 0.1, a requirement that is readily satisfied in most cases.

The transition rate matrix

(1) $$Q=Q_{M}+Q_{D},$$

of dimension $\left|A|+\left(\begin{array}{c} \left|A\right|\\ 2 \end{array}\right)(N-1\right)$ is composed by mutation (*M*) and genetic drift (*D*). The only off-diagonal, nonzero entries of $Q_{M}$ are mutations away from monomorphic states

(2) $${\left(Q_{M}\right)}_{\left\{a\}\to \{(N-1)a|1b\right\}}=q_{\mathrm{ab}},$$

where the *q_ab_* are mutation rates defined by an underlying mutation model such as the HKY mutation model (Hasegawa et al. 1985), or the GTR mutation model (Tavaré 1986). Drift rates are nonzero for neighboring states only (1≤i≤N−1)

(3) $${\left(Q_{D}\right)}_{\left\{\mathrm{ia}|(N-i)b\}\to \{(i\pm 1)a|(N-i\mp 1)b\right\}}=\frac{i(N-i)}{N}.$$

Diagonal elements of $Q_{M}$ and $Q_{D}$ are set such that all row sums are zero.

### Big Data

For the simulation of large data sets, we employed a pipeline that follows (Schrempf et al. 2016). First, ten species trees with 100 leaves were randomly generated under the Yule birth model (Yule 1925). Each of the ten species trees is referred to as one of ten replicates. The height of the species trees measured in number of generations was 6 times the effective population size which is assumed constant. The Yule birth rate was set such that the expected number of species for the given height is 100. Second, for each replicate, 1,000 gene trees were simulated under the multispecies coalescent model. SimPhy (Mallo et al. 2016) was used for these steps. Finally, for each gene tree, DNA sequences with 1,000 sites were generated with Seq-Gen (Rambaut and Grassly 1997) under the HKY mutation model (Hasegawa et al. 1985). The transition to transversion ratio was κ=6.25, the stationary nucleotide frequencies were $\pi_{A}=0.3,\;\pi_{C}=0.2,\;\pi_{G}=0.2$, and $\pi_{T}=0.3$. The simulated sequences had a heterozygosity of 0.005 which is approximately four times the value observed in primates (e.g., Prado-Martinez et al. 2013).

Estimation of the original species trees with 100 leaves was performed with IQ-TREE using either PoMo or a standard concatenation method. The HKY model was used for both methods. The discretization parameter of the multivariate boundary mutation model was *N *=* *10 and weighted binomial sampling was used. Command lines for simulation and analysis are in supplementary Section S1, Supplementary Material online. The accuracy of the inferences was measured by comparing the estimated to the original species trees. Trees were normalized to a tree height of 1.0. The branch score distance and the Robinson–Foulds distance were used.

### High Heterozygosity

For the assessment of the accuracy of tree inference for different heterozygosity levels (fig. 3) the same simulation pipeline was used. In contrast to above, the height of the species trees was 3 times the effective population size, the number of taxa was twelve, and the heterozygosity values were 0.01, 0.05, and 0.1, respectively.

### Simulator

Although the above pipeline corresponds to the way how methods based on the multispecies coalescent model perform inference, estimates of PoMo are accurate (fig. 3 and Schrempf et al. 2016). When considering mutation rate heterogeneity, we have to keep in mind that the mutation rates of the multivariate boundary mutation model are not independent of the effective population size. A mutational event {a}→{(N−1)a|1b}, a,b∈A, a≠b, corresponds to a mutation in the real population with subsequent frequency shift to a value of 1/N. Of course, the probability of such an event depends on the effective population size of the real population. As a result, higher mutation rates in the multivariate boundary mutation model, associated with a higher probability to be in a polymorphic state when species split, lead to a higher probability of ILS.

In contrast, the probability of ILS derived by the multispecies coalescent model does not explicitly depend on the mutation rate. For example, a rooted, three-taxon tree has a probability of ILS proportional to $e^{-c\Delta t/N_{e}}$ (e.g., Nei 1987), where *c* is a constant, Δt is the branch length of the internal branch measured in number of generations, and *N_e_* is the effective population size. These considerations urged us to develop a multivariate boundary mutation model simulator for direct assessment of the accuracy of mutation rate heterogeneity inference.

### Advanced Models

Heterogeneity in evolutionary rates across sites may be modeled with a parametric distribution such as the Γ distribution (Yang 1994). For the multivariate boundary mutation model, complications arise because the transition rate matrix contains contributions from mutations $Q_{M}$ as well as frequency shifts due to random genetic drift $Q_{D}$. A general scaling of the transition rate matrix, like it is usually done when using Γ rate heterogeneity with substitution models, would erroneously (de)accelerate both processes. This difficulty was overcome by employing the concept of mixture models.

In brief, *K* mutation rate categories with rate modifiers *r_k_* and corresponding probabilities 1/K of a site belonging to a category are defined. The total likelihood of a mutation model accounting for mutation rate heterogeneity *M_h_* and species tree *T* given data *D* is

(4) $$L(M_{h},T|D)=\mathrm{Pr}(D|M_{h},T)=\sum_{k=1}^{K}\frac{1}{K}\mathrm{Pr}(D|Q_{k},T),$$

where

(5) $$Q_{k}=r_{k}Q_{M}+Q_{D}.$$

Hereby, the mutation rates modifiers *r_k_* are the means of the mutation rate categories. The latter is similar to the classical treatment using Γ rate heterogeneity, however, this setup allows modeling of mutation rate heterogeneity with any free mutation rate categories. We chose to use a parameterized Γ distribution because it is most used. Naturally, the usual efficiency of Γ rate heterogeneous models is not retained, because the transition matrices for different mutation rate categories are intrinsically different. That is, an analysis with *k* categories requires eigendecomposition of *k* transition rate matrices.

The accuracy in estimating the shape parameter of the Γ distributed mutation rate heterogeneity was assayed using bmm-simulate. Species trees were randomly sampled from a Yule process. The tree height measured in average number of substitutions per site was *h *=* *0.01. The speciation rate *λ* was set such that *n *=* *12 species are present on average. That is (e.g., Kendall 1949)

(6) $$\lambda =\frac{1}{h}\left(\sum_{i=1}^{n}\frac{1}{i}-1\right).$$

For each sampled species tree, sequences were simulated under the discrete multivariate boundary mutation model. The discretization parameter *N* was set to 10, and the heterozygosity to 0.0025. Similar to the big data analysis above, the HKY mutation model was used with κ=6.25 and stationary nucleotide frequencies $\pi_{A}=0.3,\;\pi_{C}=0.2,\;\pi_{G}=0.2$, and $\pi_{T}=0.3$. Four Γ mutation rate categories were used and one million sites were simulated. Ten replicate analyses with different, randomly sampled species trees were performed. The shape parameter *α* of the Γ distributed mutation rate heterogeneity was set to 0.1, 0.3, 0.5, 1.0, 5.0, and 10.0. Command lines for simulation and analysis with rate heterogeneity are in supplementary Section S2, Supplementary Material online.

### Sampling

Interpretation of data is mostly predetermined when using phylogenetic substitution models, because at the leaves of the tree, an observed character *C* can directly be mapped to a character of the alphabet A of the used substitution model. For example, the likelihood L of a character a∈A at this site and leaf is

(7) $$L(a|C)=\{\begin{array}{cc} 1.0\;\; & \;\text{if}\;a\text{=}C,\\ 0\;\; & \;\text{otherwise}. \end{array}$$

Special handling is required when encountering an unknown character *N* (not to be confused with the discretization parameter). Then, the likelihood is

(8) L(a|N)=1.0  ∀a∈A,

because all characters have the same probability of leading to the observed character *N*.

Equivalently, we designed various strategies to initialize the likelihoods of all multivariate boundary model states at the leaves of the tree. The simplest strategy is to binomially sample *N* alleles from the observed data and initialize the leaf likelihood to

(9) $$L(a|C)=\{\begin{array}{cc} 1.0\;\; & \;\text{if}\;a\text{=}\;\text{sampled}\;\text{state}\;,\\ 0\;\; & \;\text{otherwise}, \end{array}$$

similar to equation (7). We refer to this strategy as *sampled*.

We can also initialize the leaf likelihoods to the probabilities of leading to the observed data, similar to handling unknown sites with substitution models in equation (8). Assuming binomial sampling, and when observing *M* alleles at the considered site and leaf (0≤i≤N; 0≤j≤M; a,b,c,d∈A) the likelihood is

(10) $$\begin{array}{cc} & L(\{\mathrm{ia}|(N-i)b\}|\{\mathrm{jc}|(M-j)d\})\\ & =\{\begin{array}{cc} \left(\begin{array}{c} M\\ j \end{array}\right){\left(\frac{i}{N}\right)}^{j}{\left(\frac{N-i}{N}\right)}^{M-j}\;\; & \;\text{if}\;a\text{=}c\;\text{and}\;b\text{=}d,\\ 0\;\; & \;\text{otherwise}. \end{array} \end{array}$$

We have termed this way of initializing likelihoods *weighted binomial* sampling. In the same manner, the likelihood with *weighted hypergeometric* sampling is initialized as

(11) $$\begin{array}{cc} & L(\{\mathrm{ia}|(N-i)b\}|\{\mathrm{jc}|(M-j)d\})\\ & =\{\begin{array}{cc} \left(\begin{array}{c} i\\ j \end{array}\right)\left(\begin{array}{c} N-i\\ M-j \end{array}\right)/\left(\begin{array}{c} N\\ M \end{array}\right)\;\; & \;\text{if}\;a\text{=}c\;\text{and}\;b\text{=}d,\\ 0\;\; & \;\text{otherwise}. \end{array} \end{array}$$

More details on the sampling strategies are given in supplementary Section S3, Supplementary Material online.

### Bootstrapping Using Real Data

We revisited the great ape data set of six species subdivided into 12 populations with up to 23 individuals each (Prado-Martinez et al. 2013). The exome-wide data set includes roughly 2.8 million, 4-fold degenerate sites. Data preparation is described in De Maio et al. (2015), the counts file is available on https://github.com/pomo-dev/data, last accessed March 11, 2019.

For the analysis, we used ModelFinder and assessed branch support with 1,000 bootstraps. We tested estimations with and without Γ mutation rate heterogeneity with four discrete categories combined with UFBoot2 and SH-aLRT. The discretization parameter was *N *=* *9. The command used was iqtree -s hg18-all.cf -alrt1000-bb1000.

## Supplementary Material

Supplementary data are available at *Molecular Biology and Evolution* online.

## Supplementary Material

Supplementary_Material_msz043

## References

[msz043-B1] Akaike H. 1973 Information theory and an extension of the maximum likelihood principle In: BN Petrov, F Csaki, editors. Proceedings of the 2nd International Symposium on Information Theory, Tsahkadsor, Armenia, USSR, September 2–8, 1971. Budapest (Hungary): Akadémiai Kiadó p. 267–281.

[msz043-B2] De Maio N, Schrempf D, Kosiol C. 2015 PoMo: an allele frequency-based approach for species tree estimation. Syst Biol. 64 6:1018–1031. 26209413 10.1093/sysbio/syv048 PMC4604832

[msz043-B3] Degnan JH. 2013 Anomalous unrooted gene trees. Syst Biol. 62 4:574–590. 23576318 10.1093/sysbio/syt023

[msz043-B4] Degnan JH, Rosenberg NA. 2009 Gene tree discordance, phylogenetic inference and the multispecies coalescent. Trends Ecol Evol. 24 6:332–340. 19307040 10.1016/j.tree.2009.01.009

[msz043-B5] Drummond AJ, Rambaut A. 2007 BEAST: Bayesian evolutionary analysis by sampling trees. BMC Evol Biol. 7 1:214. 17996036 10.1186/1471-2148-7-214 PMC2247476

[msz043-B6] Efron B. 1979 Bootstrap methods: another look at the jackknife. Ann Stat. 7 1:1–26.

[msz043-B7] Felsenstein J. 1985 Confidence limits on phylogenies: an approach using the bootstrap. Evolution 39 4:783–791. 28561359 10.1111/j.1558-5646.1985.tb00420.x

[msz043-B8] Gadagkar SR, Rosenberg MS, Kumar S. 2005 Inferring species phylogenies from multiple genes: concatenated sequence tree versus consensus gene tree. J Exp Zool B: Mol Dev Evol. 304B 1:64–74. 10.1002/jez.b.21026 15593277

[msz043-B9] Guindon S, Dufayard J-F, Lefort V, Anisimova M, Hordijk W, Gascuel O. 2010 New algorithms and methods to estimate maximum-likelihood phylogenies: assessing the performance of PhyML 3.0. Syst Biol. 59 3:307–321. 20525638 10.1093/sysbio/syq010

[msz043-B10] Hasegawa M, Kishino H, Yano T-A. 1985 Dating of the human-ape splitting by a molecular clock of mitochondrial DNA. J Mol Evol. 22 2:160–174. 3934395 10.1007/BF02101694

[msz043-B11] Heled J, Drummond AJ. 2010 Bayesian inference of species trees from multilocus data. Mol Biol Evol. 27 3:570–580. 19906793 10.1093/molbev/msp274 PMC2822290

[msz043-B12] Hoang DT, Chernomor O, von Haeseler A, Minh BQ, Le SV. 2018 UFBoot2: improving the ultrafast bootstrap approximation. Mol Biol Evol. 35 2:518–522. 29077904 10.1093/molbev/msx281 PMC5850222

[msz043-B13] Kalyaanamoorthy S, Minh BQ, Wong TKF, von Haeseler A, Jermiin LS. 2017 ModelFinder: fast model selection for accurate phylogenetic estimates. Nat Methods 14 6:587–589. 28481363 10.1038/nmeth.4285 PMC5453245

[msz043-B14] Kendall DG. 1949 Stochastic processes and population growth. JR Stat Soc Ser B (Methol). 11 2:230–282.

[msz043-B15] Knowles LL. 2009 Estimating species trees: methods of phylogenetic analysis when there is incongruence across genes. Syst Biol. 58 5:463–467. 20525600 10.1093/sysbio/syp061

[msz043-B16] Kuhner MK, Felsenstein J. 1994 A simulation comparison of phylogeny algorithms under equal and unequal evolutionary rates. Mol Biol Evol. 11 3:459–468. 8015439 10.1093/oxfordjournals.molbev.a040126

[msz043-B17] Leaché AD, Oaks JR. 2017 The utility of single nucleotide polymorphism (SNP) data in phylogenetics. Ann Rev Ecol Evol Syst. 48 1:69–84.

[msz043-B18] Liu L. 2008 BEST: Bayesian estimation of species trees under the coalescent model. Bioinformatics 24 21:2542–2543. 18799483 10.1093/bioinformatics/btn484

[msz043-B19] Liu L, Yu L, Edwards SV. 2010 A maximum pseudo-likelihood approach for estimating species trees under the coalescent model. BMC Evol Biol.10 1:302. 20937096 10.1186/1471-2148-10-302 PMC2976751

[msz043-B20] Lynch M, Ackerman MS, Gout JF, Long H, Sung W, Thomas WK, Foster PL. 2016 Genetic drift, selection and the evolution of the mutation rate. Nat Rev Genet. 17 11:704–714. 27739533 10.1038/nrg.2016.104

[msz043-B21] Maddison WP. 1997 Gene trees in species trees. Syst Biol. 46 3:523–536.

[msz043-B22] Mallo D. 2017. Evaluation of phylogenomic methods for species tree estimation [Ph.D. thesis]. Universidad de Vigo.

[msz043-B23] Mallo D, De Oliveira Martins L, Posada D. 2016 SimPhy: phylogenomic simulation of gene, locus, and species trees. Syst Biol. 65 2:334–344. 26526427 10.1093/sysbio/syv082 PMC4748750

[msz043-B24] Mirarab S, Reaz R, Bayzid MS, Zimmermann T, Swenson MS, Warnow T. 2014 ASTRAL: genome-scale coalescent-based species tree estimation. Bioinformatics 30 17:i541–i548. 25161245 10.1093/bioinformatics/btu462 PMC4147915

[msz043-B25] Moran PAP. 1958 Random processes in genetics. Math Proc Camb Philos Soc. 54 01:60–71.

[msz043-B26] Nei M. 1987 Molecular evolutionary genetics*.* New York: Columbia University Press.

[msz043-B27] Nguyen L-T, Schmidt HA, von Haeseler A, Minh BQ. 2015 IQ-TREE: a fast and effective stochastic algorithm for estimating maximum-likelihood phylogenies. Mol Biol Evol. 32 1:268–274. 25371430 10.1093/molbev/msu300 PMC4271533

[msz043-B28] Pamilo P, Nei M. 1988 Relationships between gene trees and species trees. Mol Biol Evol. 5 5, 568–583. 3193878 10.1093/oxfordjournals.molbev.a040517

[msz043-B29] Prado-Martinez J, Sudmant PH, Kidd JM, Li H, Kelley JL, Lorente-Galdos B, Veeramah KR, Woerner AE, O’Connor TD, Santpere G, et al 2013 Great ape genetic diversity and population history. Nature 499 7459:471–475. 23823723 10.1038/nature12228 PMC3822165

[msz043-B30] Rambaut A, Grassly NC. 1997 Seq-Gen: an application for the Monte Carlo simulation of DNA sequence evolution along phylogenetic trees. Comput Appl Biosci. 13 3:235–238. 9183526 10.1093/bioinformatics/13.3.235

[msz043-B31] Rannala B, Yang Z. 2003 Bayes estimation of species divergence times and ancestral population sizes using DNA sequences from multiple loci. Genetics 164 4:1645–1656. 12930768 10.1093/genetics/164.4.1645 PMC1462670

[msz043-B32] Robinson DF, Foulds LR. 1981 Comparison of phylogenetic trees. Math Biosci. 53(1–2):131–147.

[msz043-B33] Schrempf D, Hobolth A. 2017 An alternative derivation of the stationary distribution of the multivariate neutral Wright–Fisher model for low mutation rates with a view to mutation rate estimation from site frequency data. Theor Popul Biol. 114:88–94. 28041892 10.1016/j.tpb.2016.12.001

[msz043-B34] Schrempf D, Minh BQ, De Maio N, von Haeseler A, Kosiol C. 2016 Reversible polymorphism-aware phylogenetic models and their application to tree inference. J Theor Biol. 407:362–370. 27480613 10.1016/j.jtbi.2016.07.042

[msz043-B35] Schwarz GE. 1978 Estimating the dimension of a model. Ann Stat. 6 2:461–464.

[msz043-B36] Tavaré S. 1986 Some probabilistic and statistical problems in the analysis of DNA sequences. Lect Math Life Sci. 17:57–86.

[msz043-B37] Vogl C, Clemente F. 2012 The allele-frequency spectrum in a decoupled Moran model with mutation, drift, and directional selection, assuming small mutation rates. Theor Popul Biol. 81 3:197–209. 22269092 10.1016/j.tpb.2012.01.001 PMC3315028

[msz043-B38] Woodhams MD, Fernández-Sánchez J, Sumner JG. 2015 A new hierarchy of phylogenetic models consistent with heterogeneous substitution rates. Syst Biol. 64 4:638–650. 25858352 10.1093/sysbio/syv021 PMC4468350

[msz043-B39] Yang Z. 1994 Maximum likelihood phylogenetic estimation from DNA sequences with variable rates over sites: approximate methods. J Mol Evol. 39 3:306–314. 7932792 10.1007/BF00160154

[msz043-B40] Yang Z. 2006 Computational molecular evolution. Vol. 284. Oxford: Oxford University Press.

[msz043-B41] Yule GU. 1925 A mathematical theory of evolution, based on the conclusions of Dr. J. C. Willis, F.R.S. Philos Trans R Soc B Biol Sci. 213(402–410):21–87.

