# Supplementary material for: Polymorphism-Aware Species Trees with Advanced Mutation Models, Bootstrap, and Rate Heterogeneity
Source: Mol Biol Evol. 2019 Mar 2;36(6):1294–301. doi: 10.1093/molbev/msz043 (PMC6526911; doi:10.1093/molbev/msz043)
Supplement: Supplementary_Material_msz043 [file supplementary_material_msz043.zip › MBE-PoMo-Supplement.pdf]

Polymorphism-aware species trees with advanced mutation models, bootstrap and rate heterogeneity
—
Supplementary material

Dominik Schrempf[1,2,4], Bui Quang Minh[3,4,5], Arndt von Haeseler[4,6], and Carolin Kosiol[2,7]

[1]Department of Biological Physics, Eötvös Loránd University, Budapest, Hungary
[2]Centre for Biological Diversity, University of St Andrews, United Kingdom
[3]Ecology and Evolution, Research School of Biology, Australian National University, Australia
[4]Center for Integrative Bioinformatics Vienna, Max F. Perutz Laboratories, University of Vienna, Medical University of Vienna, Austria
[5]Research School of Computer Science, Australian National University, Australia
[6]Bioinformatics and Computational Biology, Faculty of Computer Science, University of Vienna, Austria
[7]Institut für Populationsgenetik, Vetmeduni Vienna, Austria

February 16, 2019

Contents

S1 Big data **2**

S2 Simulator and rate heterogeneity **4**

S3 Sampling strategy **6**

S1 Big data

Command lines used for simulation and analysis pipeline.

1. Generate gene trees with **SimPhy**.

```
simphy -sb f:0.0006978962529399367 -rs 20 -rl f:1 \
  -rg 1000 -sp f:1000 -sg f:1 -sl f:100 -st f:6000 \
  -si f:10 -su f:0.001 \
  -o Yule100Tree_6NeHeight_10Indivs_1000Genes
```

2. Generated gene trees need to be extracted into separate files (e.g. with Python, not shown here).

3. Generate multi-sequence alignment for each gene tree using **SeqGen**.

```
seq-gen -mHKY -f0.3,0.2,0.2,0.3 -t3.0 -l1000 \
  -n1 -on -s0.0025
  < Yule100Tree_6NeHeight_10Indivs_Tree_Gene1 \
  > Yule100Tree_6NeHeight_10Indivs_MSA_Gene1
```

4a. Concatenation of all genes (e.g. with Python, not shown here). Subsequent analysis with concatenation method.

```
iqtree -m HKY \
  -s Yule100Tree_6NeHeight_10Indivs_MSA_AllGenes
```

4b. Preparation of counts files (e.g. with `cflib` from `https://github.com/pomo-dev/cflib`, not shown here). Subsequent analysis with IQ-TREE PoMo.

```
iqtree -m HKY+P+N10+WB \
  -s Yule100Tree_6NeHeight_10Indivis_CountsFile_AllGenes
```

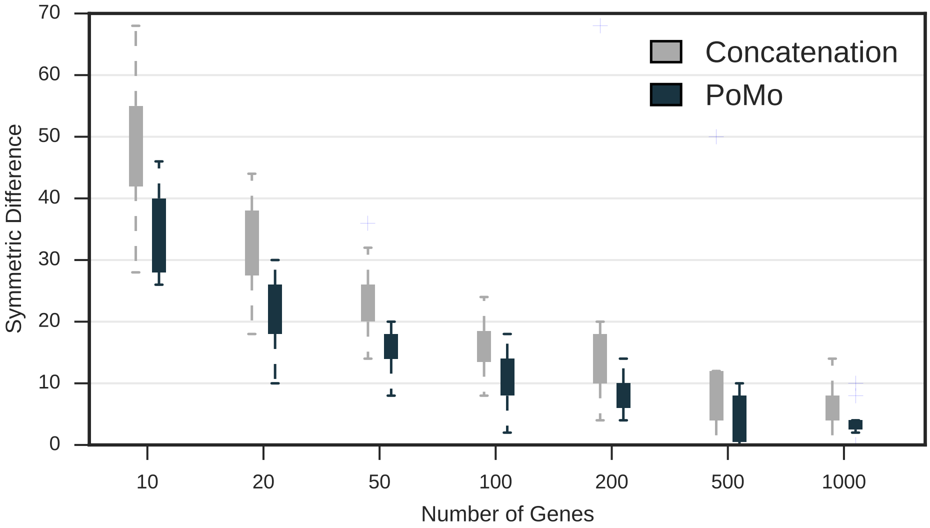

Figure S1: Symmetric difference or Robinson-Foulds metric (Robinson and Foulds, 1981) of concatenation approach and IQ-TREE PoMo with $N = 10$ and weighted binomial sampling for the Yule trees with 100 species and 10 samples each. The tree height is $6N_e$. The HKY model was used for both inference methods. The heterozygosity is $\theta_W = 0.005$ per site. Each gene spans 1000 sites. The error bars are standard deviations of ten replicate analyses.

S2 Simulator and rate heterogeneity

Command lines used for simulation and analysis pipeline.

1. For example, generate counts files with rate heterozygosity with Γ shape parameter $\alpha = 1.0$.

```
bmm-simulate -N 10 --heterozygosity 0.0025 \
  --tree-type Yule --tree-height 0.01 \
  --tree-yule-rate 210.3210678210678 \
  --gamma-shape 1.0 --gamma-ncat 4 \
  --nsites 1000000 -o data.cf
```

2. Analysis with HKY model, rate heterogeneity, $N = 10$, and weighted hypergeometric sampling.

```
iqtree -s data.cf -m HKY+P+G4+N10+WH
```

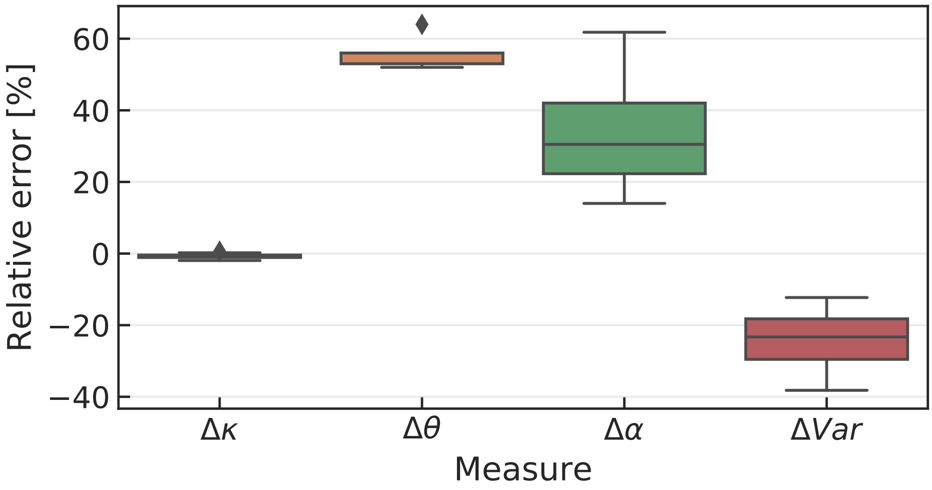

Figure S2: Relative errors of the transition to transversion ratio $\Delta\kappa$, the heterozygosity $\Delta\theta$, the shape parameter of the Γ distributed mutation rate heterogeneity $\Delta\alpha$, and the variance of the associated Γ distribution for a Yule tree of height 0.01 average number of substitutions per site and an average number of twelve species with ten samples each. One million sites where analyzed, the true heterozygosity and shape parameter are $\theta = 0.0025$ and $\alpha = 0.1$, respectively.

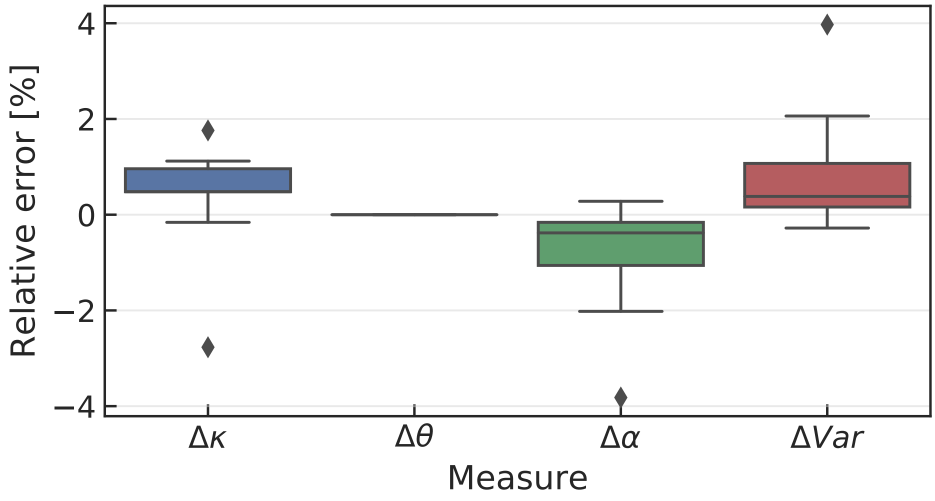

Figure S3: Relative errors of the transition to transversion ratio $\Delta\kappa$, the heterozygosity $\Delta\theta$, the shape parameter of the Γ distributed mutation rate heterogeneity $\Delta\alpha$, and the variance of the associated Γ distribution for a Yule tree of height 0.01 average number of substitutions per site and an average number of twelve species with ten samples each. One million sites where analyzed, the true heterozygosity and shape parameter are $\theta = 0.0025$ and $\alpha = 0.5$, respectively.

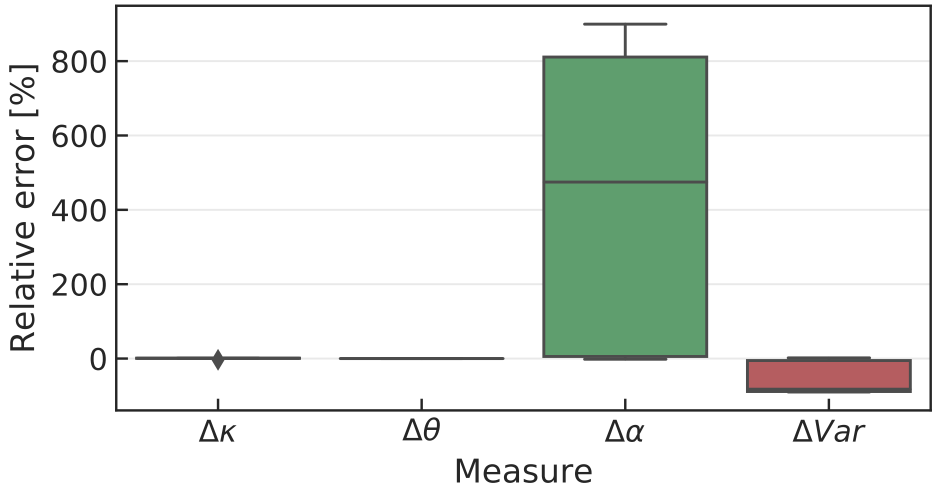

Figure S4: Relative errors of the transition to transversion ratio $\Delta\kappa$, the heterozygosity $\Delta\theta$, the shape parameter of the Γ distributed mutation rate heterogeneity $\Delta\alpha$, and the variance of the associated Γ distribution for a Yule tree of height 0.01 average number of substitutions per site and an average number of twelve species with ten samples each. One million sites where analyzed, the true heterozygosity and shape parameter are $\theta = 0.0025$ and $\alpha = 10$, respectively.

S3 Sampling strategy

Interpretation of data is mostly predetermined when using phylogenetic substitution models, because at the leaves of the tree, observed states can directly be mapped to internal states of the used substitution model. Let the state space of the considered substitution model be nucleotide bases $\mathcal{A} = \{A, C, G, T\}$. If, at a specific site in the alignment and leaf of the tree, we observe an A, the likelihood vector will be set to ($a \in \mathcal{A}$)

$$\mathcal{L}(a|A) = \begin{cases} 1.0 & \text{if } a = A, \\ 0 & \text{otherwise.} \end{cases} \tag{S1}$$

Special handling is required when encountering an unknown site (usually denoted by N, not to be confused with the discretization parameter of the multivariate boundary mutation model). Then, likelihoods are initialized in an inverse manner. That is, the probability of a specific state leading to the observed data is considered. In this example, the likelihood is set to 1.0, irrespective of the nucleotide. I.e., any state at the tip is equally likely because all have the same probability of leading to the observed data

$$\mathcal{L}(a|N) = 1, \quad a \in \mathcal{A}. \tag{S2}$$

Equivalently, we would like to initialize the likelihoods of all multivariate boundary mutation model states at a specific site in the alignment and leaf of the tree. If the number of samples coincides with the discretization parameter N, and if not more than two alleles are present, the observed data is a multivariate boundary mutation model state. Like above with substitution models, the likelihood of this state can be set to 1, and the likelihood of all other states to 0. Let us suppose that $N = 10$ and that we observe 4 individuals with an A and 6 individuals with a C. Then, $x \in \mathcal{A}_{BM}$, where $\mathcal{A}_{BM}$ is the state space of the multivariate boundary mutation model

$$\mathcal{L}(x|\{4A|6C\}) = \begin{cases} 1.0 & \text{if } x = \{4A|6C\}, \\ 0 & \text{otherwise.} \end{cases} \tag{S3}$$

However, if the number of samples does not match the discretization parameter N, and this will often be the case, we have to think about other means of initializing the likelihoods of the multivariate boundary mutation model states Schrempf *et al.* (2016). The easiest one is to randomly draw N alleles with replacement from the data (each allele has equal probability of being chosen; binomial sampling). We refer to this methods as *sampled*. Let us suppose that we observe that 13 individuals have an A and 18 individuals have a C, i.e., $\{13A|18C\} \notin \mathcal{A}_{BM}$. So, we randomly pick N alleles from the observed data. Let us suppose that $N = 10$ and that we pick 3 individuals

with an A and 7 individuals with a C. So ($x \in \mathcal{A}_{BM}$)

$$\mathcal{L}(x|\{3A|7C\} \text{ sampled from } \{13A|18C\}) = \begin{cases} 1.0 & \text{if } x = \{3A|7C\}, \\ 0 & \text{otherwise.} \end{cases} \tag{S4}$$

A more canonical way is to initialize likelihoods similar to handling unknown sites with substitution models. For a specific multivariate boundary mutation model state at the considered site and leaf, the likelihood is initialized to the probability of observing the data conditioned on this state. There are many ways of defining this probability. The simplest is, (1) assume that all alleles have equal probability of being drawn, and (2) sample with (binomial) or without (hypergeometric) replacement. We do this for all multivariate boundary mutation model states. We have termed these ways of initializing likelihoods *weighted binomial* or *weighted hypergeometric* sampling.

For example, let the observed data be the same as before $\{13A|18C\}$, and let us use weighted binomial sampling. Then, the likelihood of multivariate boundary mutation model state $\{1A|9C\}$ would be

$$\mathcal{L}(\{1A|9C\}|\{13A|18C\}) = \Pr(\{13A|18C\}|\{1A|9C\}) \tag{S5}$$

$$= \binom{31}{13}\left(\frac{1}{10}\right)^{13}\left(\frac{9}{10}\right)^{18}. \tag{S6}$$

In general, when observing M alleles at the considered site and leaf ($0 \leq i \leq N$; $0 \leq j \leq M$; $a, b, c, d \in \mathcal{A}$)

$$\mathcal{L}(\{ia|(N-i)b\}|\{jc|(M-j)d\}) = \begin{cases} \binom{M}{j}\left(\frac{i}{N}\right)^j\left(\frac{N-i}{N}\right)^{M-j} & \text{if } a = c \text{ and } b = d, \\ 0 & \text{otherwise.} \end{cases} \tag{S7}$$

We implemented weighted hypergeometric sampling, because weighted binomial sampling leads to an overestimation of heterozygosity. The reason is that, if we observe a monomorphic site, polymorphic and monomorphic multivariate boundary mutation model states have non-zero likelihood; but if we observe a polymorphic site, only polymorphic multivariate boundary mutation model states have a non-zero likelihood. Weighted hypergeometric sampling does not have this property, and retains the heterozygosity. In this respect, it is the better method, but from our numerous simulations we see that, in general, weighted binomial sampling has lower errors in terms of branch score distance.

References

Robinson, D. F. and Foulds, L. R. 1981. Comparison of phylogenetic trees. *Mathematical Biosciences*, 53(1-2): 131–147.

Schrempf, D., Minh, B. Q., De Maio, N., von Haeseler, A., and Kosiol, C. 2016. Reversible polymorphism-aware phylogenetic models and their application to tree inference. *Journal of Theoretical Biology*, 407: 362–370.
