# Supplementary figures and images for: Polymorphism-Aware Species Trees with Advanced Mutation Models, Bootstrap, and Rate Heterogeneity
Source: Mol Biol Evol. 2019 Mar 2;36(6):1294–301. doi: 10.1093/molbev/msz043 (PMC6526911; doi:10.1093/molbev/msz043)

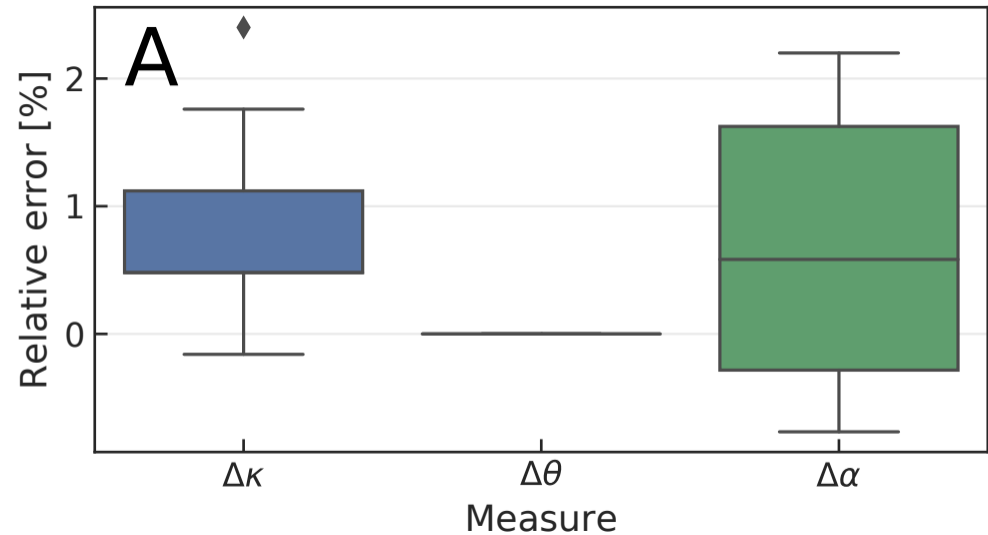

Supplement: Supplementary_Material_msz043 [file supplementary_material_msz043.zip › 0-30Ga4Categ-A.pdf]

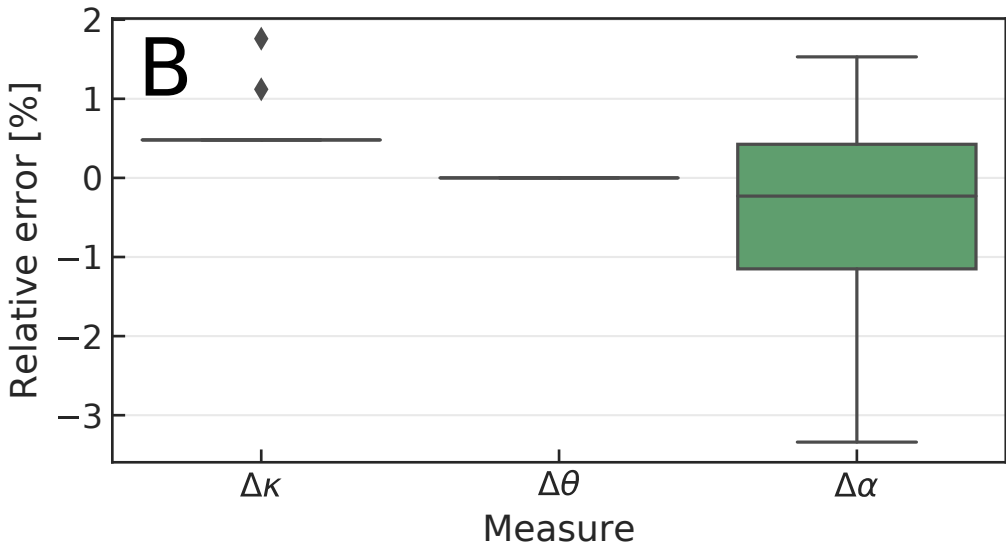

Supplement: Supplementary_Material_msz043 [file supplementary_material_msz043.zip › 1-00Ga4Categ-B.pdf]

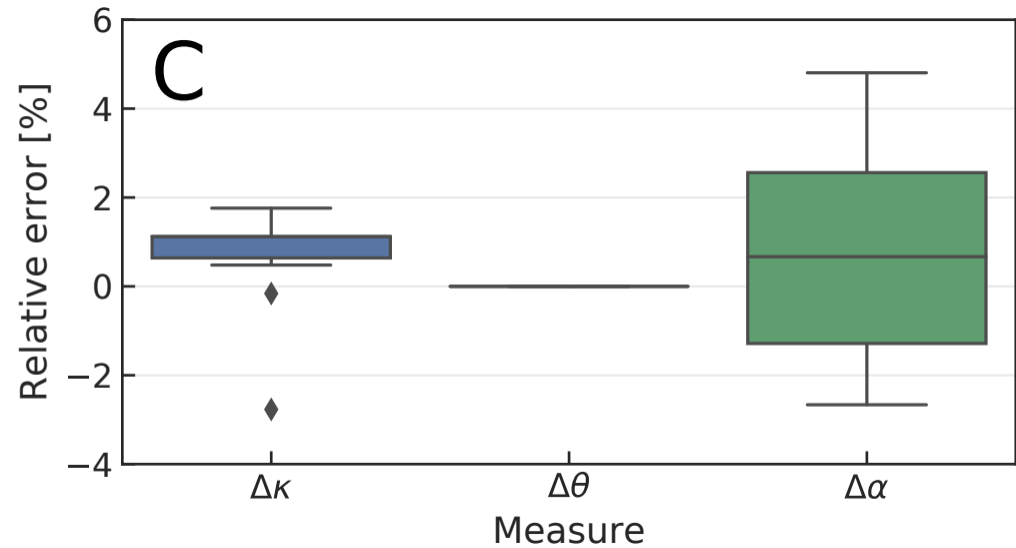

Supplement: Supplementary_Material_msz043 [file supplementary_material_msz043.zip › 5-00Ga4Categ-C.pdf]

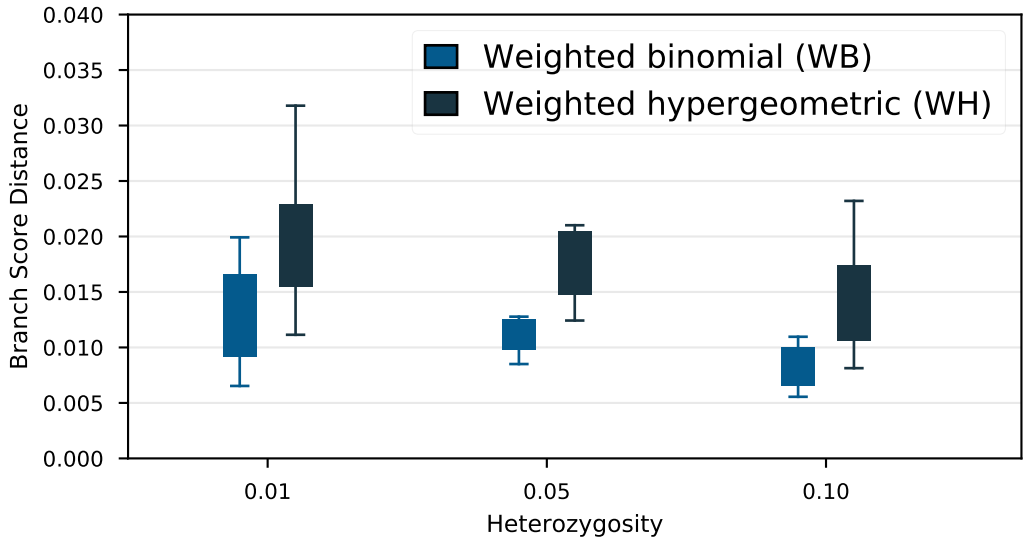

Supplement: Supplementary_Material_msz043 [file supplementary_material_msz043.zip › bsds_heterozygosity.pdf]

Branch Score Distance

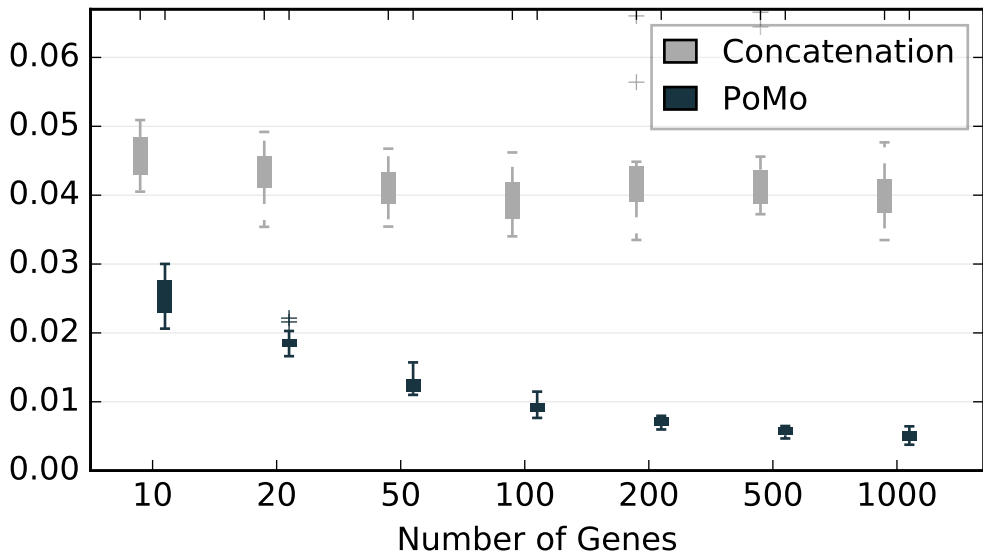

Supplement: Supplementary_Material_msz043 [file supplementary_material_msz043.zip › distance_y100_6Ne_10S_10N.pdf]

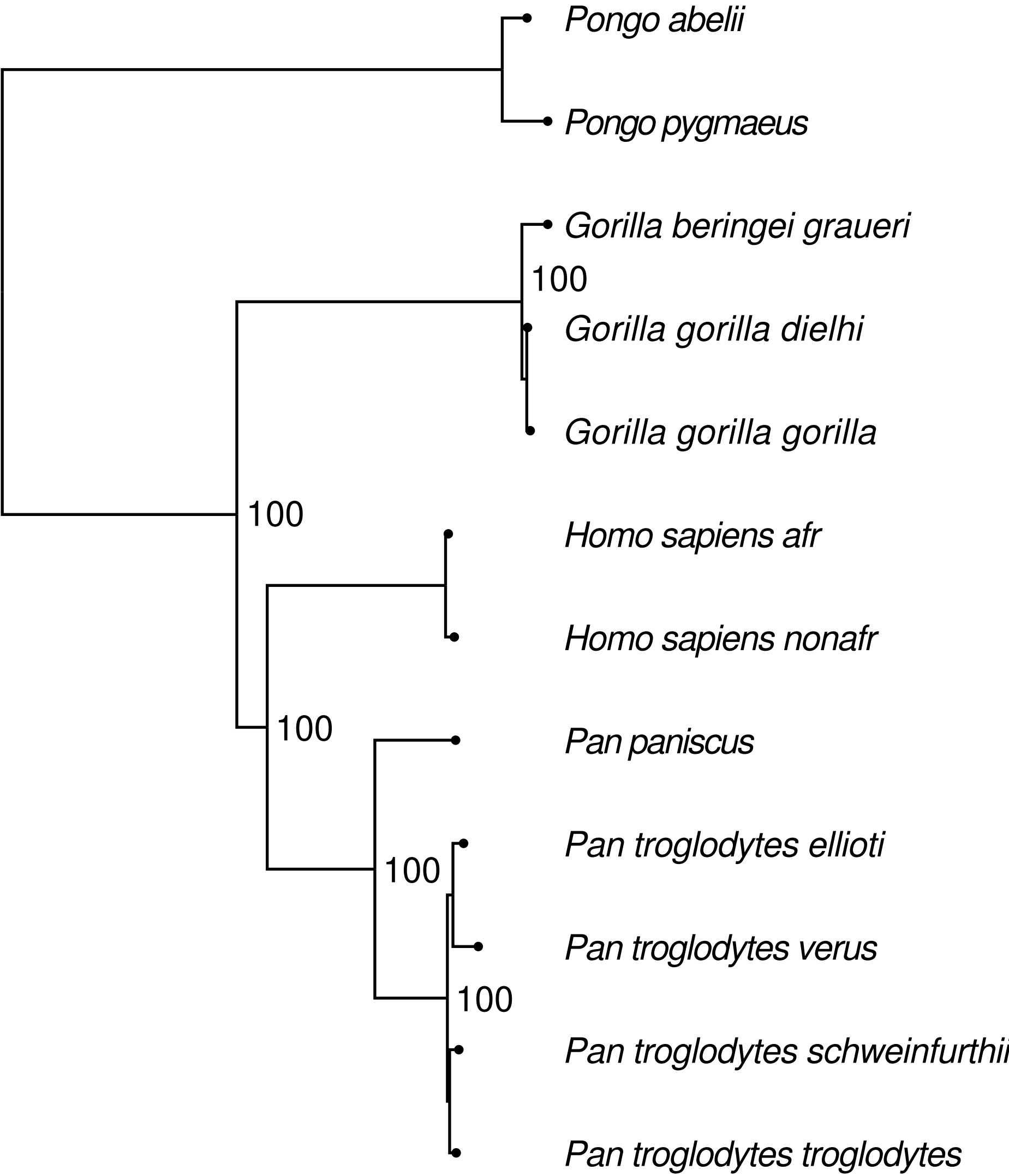

0.2

Supplement: Supplementary_Material_msz043 [file supplementary_material_msz043.zip › primates_tree.pdf]
